# Efficiently Multi-User Searchable Encryption Scheme with Attribute Revocation and Grant for Cloud Storage

**DOI:** 10.1371/journal.pone.0167157

**Published:** 2016-11-29

**Authors:** Shangping Wang, Xiaoxue Zhang, Yaling Zhang

**Affiliations:** 1 School of Science, Xi’an University of Technology, Xi’an, Shaanxi, China; 2 School of Computer Science and Engineering, Xi’an University of Technology, Xi’an, Shaanxi, China; West Virginia University, UNITED STATES

## Abstract

Cipher-policy attribute-based encryption (CP-ABE) focus on the problem of access control, and keyword-based searchable encryption scheme focus on the problem of finding the files that the user interested in the cloud storage quickly. To design a searchable and attribute-based encryption scheme is a new challenge. In this paper, we propose an efficiently multi-user searchable attribute-based encryption scheme with attribute revocation and grant for cloud storage. In the new scheme the attribute revocation and grant processes of users are delegated to proxy server. Our scheme supports multi attribute are revoked and granted simultaneously. Moreover, the keyword searchable function is achieved in our proposed scheme. The security of our proposed scheme is reduced to the bilinear Diffie-Hellman (BDH) assumption. Furthermore, the scheme is proven to be secure under the security model of indistinguishability against selective ciphertext-policy and chosen plaintext attack (IND-sCP-CPA). And our scheme is also of semantic security under indistinguishability against chosen keyword attack (IND-CKA) in the random oracle model.

## I. Introduction

The fuzzy identity based encryption (IBE) which is regarded as the prototype of attribute-based cryptography was put forward by Sahai and Waters [1] in 2005. In an attribute-based encryption system, each user has a number of descriptive attributes (such as gender, age, education, occupation, etc.). Meanwhile, the users’ private key and ciphertext are link with some described attribute set and access strategy respectively. When the private key is matched with ciphertext, the user can decrypt the ciphertext.

Goyal et al. [2] put the ABE scheme into CP-ABE scheme and the key-policy attribute-based encryption (KP-ABE) scheme, and definitions are given respectively.

Bethencourt et al. [3] provided a new structure. The scheme can not only achieve a flexible access structure but also has an important characteristic of anti-collusion. That is, different users can not add their own access right by collusion their private key. Besides, there are some other outstanding articles such as the scheme proposed by Emura et al. [4] which has a certain contribution to the computational complexity and storage load.

The above-mentioned CP-ABE schemes have made outstanding contributions, but due to the constant changes of the realistic situation, the schemes still face new challenges. Once some users' attributes change, the system should timely update these users' attribute set and the corresponding private key.

A number of programs research about attribute revocation have been put forward [5–13]. Generally speaking, the revocation mechanism can be divide into two types: direct revocation scheme [6–9] and indirect revocation scheme [10–13]. The big difference between them is that the direct revocation scheme is enforced by a specified revocation list and indirect revocation scheme is enforced by updating the private key of the non-revoked users (Implicitly, the revoked users' private key are revoked). Zhang et al. [8] put forward a scheme with direct revocation, which characterized by the fact that the length of the encrypted text is fixed, and the partial ciphertext update is only required when the revocation occurs. The scheme put forward by Yu et al. [10] achieves an efficient encryption update through proxy re encryption. But there is a limit to the scheme. That is the fixed strategy. Due to the high efficiency and the limitation of the scheme, Naruse et al. [13] made a further study of this article. The scheme proposed by them can be applied to a more flexible access strategy.

On the other hand, due to the continuous development of computer network and the outsourcing technology many enterprises began to establish their own local network and database. Through the establishment of a certain data encryption and an access control, they passed their database to a third party management. Since the third party is not credible, efficient search capability and secure search process are two important tasks in the present study. Some articles research on these two directions have been put forward [14–17].

Bao at al. [14] put forward a scheme which can be applied to the cloud storage environment. This program can realize the multi user search process. Because of the users’ access rights in the system are different according to their own attribute set. The efficiency of system should be further improved with the increase of users’ number.

Some schemes research on highly efficient access control of multi user keyword search have been put forward [18–24].

Recently, Lv et al. [18] proposed an efficient keyword searchable model. However, the scheme does not have a complete security model. When a user’s attributes in the system change, the limitations of the program appeared. Kaci et al. [23] put forward a scheme that is consistent with ACAS (Access Control Aware Search) principle and improve the level of confidentiality of outsourced data. Nonetheless, the efficiency of the proposed model is evaluated according to data size.

Most of the existing multi user attribute-based keyword searchable encryption schemes focus on efficient access control and fast search process, of which there are some articles can achieve revocation of user, for example by removing user’s search key in proxy server to achieve revocation [18].

In addition, some research on the security of information under specific scenarios are also proposed. Specifically, a first research direction focuses on the security of Vehicular and hoc network [25–27]. A second research direction deal with the security communication in Internet of Things (IOT) networks [28, 29]. There are other research directions, such as file search in unstructured P2P (peer-to-peer) gird networks [30–32] and WSNs (wireless sensor networks) in healthcare applications [33] etc.

### A. Our Contributions

- Our scheme supports user’s multiple attributes revocation and grant simultaneously by adding a series of attribute parameters. The attribute revocation in our scheme is a fine grain method. That is, our revocation is able to revoke some users’ some attributes, rather than to revoke a single attribute or revoke attributes in the system. The attribute grant method is similarly. In addition, the proposed scheme is proven to be IND-sCP-CPA secure.
- We use a lazy revocation technique [34] for user’s attribute and private key update process. It is to say that only when user accesses the encrypted files, it helps to update the user's attribute and private key.
- As keyword searchable process in [18] does not have a complete security proof. By changing the operation of search trapdoor in [18], we have proved that our proposed keyword searchable scheme is IND-CKA secure in the random oracle model under bilinear Diffie-Hellman (BDH) assumption.
- The function of revocation of user identity in our scheme is consistent with that in [18].

### B. Comparisons

We compare the function of our scheme with the existing schemes presented in [3, 13, 18] in Table 1.

**Table 1:** Comparisons of Our Scheme with the Main References.

scheme	access control	keyword searchable	attribute revocation for some user	attribute grant for some user	user revocation	lazy revocation
[3]	LSSS	✘	✘	✘	✘	✘
[18]	access tree	✓	✘	✘	✓	✘
[13]	LSSS	✘	✓	✓	✘	✘
Ours	access tree	✓	✓	✓	✓	✓

✓: The scheme has the function.

✘: The scheme does not have this function.

## II. Preliminaries

### A. Mathematical Tools

We first give some of the mathematical tools will be used later in this article, the specific argument can be found in the references.

**Definition 1** (Bilinear Map [2]). The definition of the two multiplication of group $G_{1}$ and $G_{2}$, so that their order is *p* and the generator of $G_{1}$ is *g*. A bilinear map $e:G_{1}\times G_{1}\to G_{2}$, which satisfies:

- *Bilinearity*: for all $u,\;v\in G_{1}$ and $a,\;b\in {\mathbb{Z}}_{p},\;e\left(u^{a},v^{b}\right)=e{\left(u,v\right)}^{ab}$;
- *Non-degeneracy*: *e*(*g*, *g*) ≠ 1;
- *Computability*: for all $u,\;v\in G_{1}$, *e*(*u*, *v*) is efficiently computable.

**Definition 2** (Lagrange Coefficient [24]). The definition of a Lagrange coefficient is Δ_*i*,*S*_(*x*), which $i\in {\mathbb{Z}}_{p}$ and the elements of set *S* belong to ${\mathbb{Z}}_{p}$. Then we have the following equation:

$$\Delta_{i,S}\left(x\right)=\prod_{j\in S,j\neq i}\frac{x-j}{i-j}$$

### B. Access Tree

In this paper, we use the access tree as the access policy.

**Definition 3** (Access Tree [18]). In the access tree, the number of child nodes of each interior node *x* is denoted as *num*_*x*_. The threshold value of each node is defined as (*k*_*x*_, *num*_*x*_), which is 0 < *k*_*x*_ ≤ *num*_*x*_. In particular, when *k*_*x*_ = 1 threshold for an ′OR′ gate. When *k*_*x*_ = *num*_*x*_ for an ′AND′ gate. Furthermore, each leaf node are correlated and attribute. For the convenience of using access tree, we define several functions as follow.

- *parent*(*x*): this returns the parent node of a node *x* except the root node.
- *index*(*x*): assuming that the children nodes of each node are numbered from 1 to *num*, this returns such a number associated with the node *x*.
- *att*(*x*): this returns the attribute associated with a leaf node *x*.

### C. BDH Problem

Choose two cyclic group $G_{1}$ and $G_{2}$, enable their order is *p*. And a map $e:G_{1}\times G_{1}\to G_{2}$ is a valid bilinear map. BDH problem under the tuple $<g,\;G_{1},\;G_{2},\;e>$ can be defined as: fix a generator *g* of $G_{1}$, as well as *g*^*a*^, *g*^*b*^, *g*^*c*^ for some random $a,\;b,\;c\in {\mathbb{Z}}_{p}$, compute $e{\left(g,g\right)}^{abc}\in G_{2}$.

*BDH assumption* [35]. The assumption is valid if there is no polynomial-time adversary can be non-negligible probability to solve the above BDH problem.

## III. System and Security Models

### A. System Model

**Fig 1:**
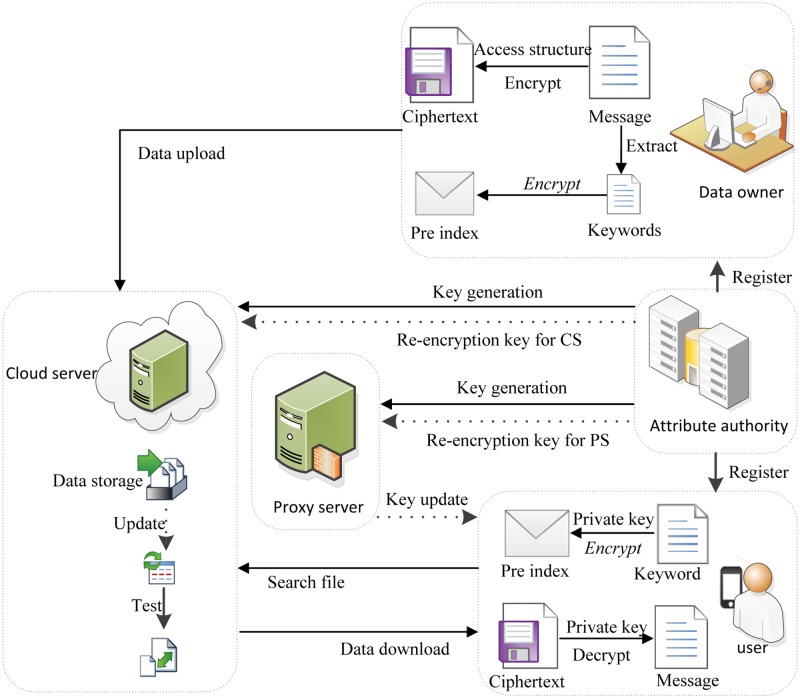
System model of the proposed scheme.

First, define five entities of the system: an attribute authority, dada owners, a proxy server, a cloud sever, users, can be described below. The system model of our scheme is given in Fig 1.

- **Attribute authority (AA).** Attribute authority is entirely credible to other entities and is responsible for the system establishment, new user register, attributes assignment and key generation. When some users’ attribute set change (that is, some attributes are revoked or granted), AA establishes a revoked and a granted user list set for each attribute respectively and updates the public parameter, master key, proxy update key and proxy grant key.
- **Data owner (DO).** Data owner is responsible for uploading all the data files to cloud server. In order to ensure other legitimate users of the system can search for the corresponding file through the keyword, data owner needs to extract keywords and establish keyword indexes. Finally, along with the encrypted files upload to cloud server.
- **User (U).** Legitimate users can download their interest files from the system. In order to hide the search keyword, the user generates a search trapdoor. And then sends his unique identity, attribute set, partial private key component to the proxy server for updating attribute set and private key component. After receiving the updated attribute set and private key component, he sends his trapdoor together with his unique identity to cloud server. Without revealing any information about the content of the file, proxy server to help complete most of the decryption work. And then the final message is calculated by the user.
- **Proxy server (PS).** Proxy server is deployed by AA. It re-encrypts encrypted shared data and updats user's attribute set and corresponding private key by using the proxy update key and proxy grant key received from AA. It also can help the users execute most CP-ABE decryption task.
- **Cloud server (CS).** This paper mainly use the large storage characteristics of CS to store the data files in the system. Besides, it also helps to generate keyword index and trapdoor. In order to achieve efficient search we use the *D*. *Data Upload* method in [18] to store and search files. Also similar to the *G*. *User Revocation* method in [18], CS can perform user revocation operation.

### B. Algorithms Definitions

Our proposed efficiently multi-user searchable encryption scheme with attribute revocation and grant for cloud storage is composed of thirteen randomized polynomial time algorithms.

- **AA.Setup** (*λ*, *U*)→(*PP*, *MK*, *UK*, *GK*): The setup algorithm takes a security parameter *λ* and an attribute universe description *U* as input. It outputs the public parameters *PP*, master private key *MK*, proxy update key *UK* and proxy grant key *GK*.
- **DO.Enc** (PP,M,T)→CT: The encryption algorithm takes public parameters *PP*, a message *M*, and an access structure T over the universe of attributes as input. It generates a ciphertext *CT*.
- **AA.KenGen** $\left(MK,U_{id},S_{U_{id}}\right)\to \left(SK_{U_{id}},A_{U_{id}},B_{U_{id}}\right)$: The key generation algorithm takes master private key *MK*, a unique user identity *U*_*id*_ and the corresponding attribute set $S_{U_{id}}$ as input. It outputs *U*_*id*_’s corresponding private key $SK_{U_{id}}$, user’s search key $A_{U_{id}}$, and user’s search key $B_{U_{id}}$ in CS.
- **U.Dec** $\left(CT,SK_{U_{id}}\right)\to M$: The decryption algorithm take a ciphertext *CT*, and a private key $SK_{U_{id}}$ as input. If the set of attributes $S_{U_{id}}$ related to $SK_{U_{id}}$ satisfies the access structure T related to *CT*, then it successfully decrypt and output the message *M*
- **AA.ReKenGen** (*PP*, *MK*, *γ*, Δ*L*_*γ*_, *η*, Δ*L*_*η*,_*UK*, *GK*)→(*PP*′, *MK*′, *UK*′, *GK*′, *RKγ*): The re-encryption key generation algorithm tekes public parameters *PP*, a master key *MK*, a set of attributes *γ*, the attribute in *γ* which is to be revoked for some users, and the corresponding revoked user list Δ*L*_*γ*_, a set of attribute *η*, the attribute in *η* is to be granted for some users, and the corresponding granted user list Δ*L*_*η*_, proxy update key *UK*, and proxy grant key *GK* as input. It generates the updated public parameters *PP*′, the redefined master key *MK*′, the redefined proxy update key *UK*′, the proxy grant key *GK*′, and the re-encryption key *RK*_*γ*_.
- **CS.ReEnc** (*γ*, *C*_*γ*_, *rk*)→*RK*_*γ*_: The re-encryption algorithm takes a set of attribute *γ* which some users be revoked, the ciphertext component *C*_*γ*_ = {*C*_*x*_}_*att*(*x*)∈*γ*_ ⊂ *CT*, the re-encryption key *RK*_*γ*_ as input. It outputs the re-encryption ciphertext component $C_{\gamma }\prime ={\left\{C_{x}^{*}\right\}}_{att\left(x\right)\in \gamma }$.
- **PS.ReKey** $\left(U_{id},S_{U_{id}},ver_{U_{id}},D_{U_{id}},UK,\gamma \right)\to \left(S_{U_{id}}\prime ,ver_{U_{id}}\prime ,D_{U_{id}}\prime \right)$: The key regeneration algorithm takes a unique user identity *U*_*id*_ and the corresponding attribute set $S_{U_{id}}$, version number $ver_{U_{id}}$, the private key component $D_{U_{id}}={\left\{D_{i}\right\}}_{i\in \gamma }\subset SK_{U_{id}}$, proxy update key *UK*, a set of attributes *γ*, the attribute in *γ* which is to be revoked by some users as input. It outputs the updated user attribute set $S_{U_{id}}\prime$, version number $ver_{U_{id}}\prime$ and private key component $D_{U_{id}}\prime ={\left\{D_{i}\prime \right\}}_{i\in \gamma }\subset SK_{U_{id}}$.
- **PS.GrantAtt** $\left(U_{id},PP,GK,\eta \right)\to \left(\eta_{U_{id}},SK_{\eta_{U_{id}}}\right)$: The attribute grant algorithm takes a unique user identity *U*_*id*_, the public parameters *PP*, the proxy grant key *GK*, a set of attribute *η*, the attribute in *η* which is to be granted by some users as input. It outputs a set of attribute $\eta_{U_{id}}$ which is to be granted to the user *U*_*id*_ and the corresponding private key component $SK_{\eta_{U_{id}}}={\left\{D_{i},D_{i}^{*}\right\}}_{i\in \eta_{U_{id}}}$.
- **O.PreIndex** (*W*)→(*E*): The pre index generation algorithm for the data owner takes keyword set *W* = {*w*_1_, ⋯, *w*_*m*_} as input. It outputs data owner’s pre keywords index set *E* = (*E*_1_, ⋯, *E*_*m*_).
- **CS.Index** $\left(E,B_{U_{id}}\right)\to \left(V\right)$: The index generation algorithm for the CS takes data owner’s pre keywords index set *E* = (*E*_1_, ⋯, *E*_*m*_) and data owner’s search key in CS $B_{U_{id}}$ as input. It outputs CS’s index parameter set *V* = (*V*_1_, ⋯, *V*_*m*_).
- **O.PostIndex** $\left(V,A_{U_{id}}\right)\to \left(I_{W}\right)$: The post index generation algorithm for DO takes CS’s index parameter set *V* = (*V*_1_, ⋯, *V*_*m*_), his own search key $A_{U_{id}}$ as input. It outputs DO’s post keywords index set $I_{W}=\left(I_{w_{1}},\cdots ,I_{w_{m}}\right)$.
- **U. PreTrap** $\left(w,A_{U_{id}}\right)\to \left(T_{w}\right)$: The pre trapdoor generation algorithm for user takes a keyword *w* and his own private search key $A_{U_{id}}$ as input. It outputs user’s pre trapdoor *T*_*w*_.
- **CS. PostTrap** $\left(T_{w},B_{U_{id}}\right)\to \left(k\prime \right)$: The post trapdoor generation algorithm for CS takes user’s pre trapdoor *T*_*w*_ and user’s search key $B_{U_{id}}$ in CS as input. It outputs CS’s post trapdoor *k*′.
- **CS. Test**(*I*_*W*_, *k*′)→{1, 0}: The test algorithm for the CS takes post keywords index set *I*_*W*_ and post trapdoor *k*′ as input. If the match is successful, the output is 1. Otherwise, the output is 0.

### C. Security Definitions

Similar to most previous works, the CS is supposed to be “curious-but-honest” [13].

We consider the security model as two games between a challenger C and an adversary A.

#### Game 1 (IND-sCP-CPA security model)

The adversary A is assumed to be an outsider attracter including the receiver.

**Int.** A declares an access structure $T^{*}$.

**Setup.** C takes a security parameter *λ* and runs the **Setup** algorithm. It gives the public parameter *PP* to A and keeps the master key *MK* to itself.

**Phase 1.** A adaptively issues polynomial queries as follows.

- *private key query*. A submits an attribute set *S*, where *S* does not satisfy the access structure $T^{*}$, to C. The challenger returns the corresponding private key *SK* to A.
- *update private key query*. A is allowed to issue queries for update private key *SK* for the attribute in *γ* which is to be revoked for some users. The challenger gives the updated private key *SK*′.

**Challenge.** A submits two equal length message *M*_0_ and *M*_1_. The challenger picks a random bit *b* ∈ {0, 1} and encrypts *M*_*b*_ under $T^{*}$. The challenger gives ciphertext CT* to A.

**Phase 2.** Repeat Phase 1 adaptively.

**Guess.** A outputs a guess *b*′ of *b* and wins the game if *b*′ = *b*.

The advantage of the A in this game is defined as $\begin{array}{l} \left|\text{Pr}\left[b^{\prime }=b\right]-\frac{1}{2}\right| \end{array}$.

**Definition 4.** The proposed scheme is IND-sCP-CPA secure if there is no polynomial time A who can win the above game with non-negligible advantage.

#### Game 2 (IND -CKA security model)

The adversary A is assumed to be CS.

**Setup.** Repeat game 1’s setup adaptively.

**Phase 1.** A adaptively issues polynomial following queries.

- *H*_1_-*Query*. A can query the random oracle *H*_1_.
- *H*_2_-*Query*. A can query the random oracle *H*_2_.
- *Trapdoor Queries*. A can ask any keyword’s trapdoor.

**Challenge.** A submits two keywords *w*_*0*_ and *w*_1_ where the keywords *w*_0_ and *w*_1_’s trapdoor have not been asked by A. The challenger picks a random bit *b*∈{0, 1} and creates *w*_*b*_’s trapdoor *k* to A.

**Phase 2.** Repeated phase 1 adaptively.

**Guess.** A submits a guess *b*′ of *b*. If *b*′ = *b*, A wins the game and break our scheme.

**Definition 5.** In the random oracle model, the proposed scheme is IND-CKA secure if all polynomial time adversaries have at most a negligible advantage in the above game.

## IV. Our Proposed Scheme

### A. Detail Construction of Algorithms

AA defines the universe of attributes as *U* = {1, 2, ⋯, *n*}, the unique user identity *U*_*id*_ ∈ {0, 1}* and three hash functions:

- *H*(∙): Maps an attribute to a random element of $G_{1}$.
- *H*_1_(∙): Maps a strings in {0, 1}* to a random element of $G_{1}$.
- *H*_2_(∙): Maps a random element of $G_{2}$ to a random strings of {0, 1}^*l*^.

**AA.Setup** (*λ*, *U*) → (*PP*, *MK*, *UK*, *GK*): The setup algorithm takes security parameter *λ* and attribute universe description *U* = {1, 2, ⋯, *n*} as input. It first chooses two multiplicative cyclic groups $G_{1},G_{2}$ of prime order *p*(*p* > 2^*λ*^), and a bilinear map $e:G_{1}\times G_{1}\to G_{2}$. Then, ∀*i* ∈ *U*, it random chooses $Att_{i}\in Z_{p}^{*}$ and computes a public parameter component $T_{i}=g^{Att_{i}}\in G_{1}$. And it randomly chooses *x* ∈ *Z*_*P*_ as the search master key and three random numbers *α*, *β*, *x* ∈ *Z*_*p*_, let

$$PP=\left(G_{1},g,g^{\beta },e{\left(g,g\right)}^{\alpha },T_{1},\cdots ,T_{n}\right)$$

$$MK=\left(x,\beta ,g^{\alpha },Att_{1},\cdots ,Att_{n}\right)$$

In addition, defines the system version number *ver* ∈ *N*. The initial version number is set to *ver* = 0. Set a proxy re-encrypt key set as *rk* = {*rk*_*i*_}_*i∈U*_, and *rk*_*i*_ = {*rk*_*i*,0_, ⋯, *rk*_*i*,*ver*_} is set of the proxy re-encrypt key under different version for attribute *i*. The initial value is set to *rk*_*i*,0_ = 1.

Let *L* = {*L*_*i*_}_*i*∈*U*_ represent the revoked user list set, where revocation list *L*_*i*_ represents the users list whose attribute *i* needs to be revoked, and $L^{*}={\left\{L_{i}^{*}\right\}}_{i\in U}$ represent the granted user list set, where grant list $L_{i}^{*}$ represents the users list set to whom the attribute *i* needs to be granted. The revocation list *L*_*i*_ may be empty, which means there is no user needs to be revoked for attribute *i*. So is grant list $L_{i}^{*}$.

Finally, define a set *R* which is used to reserve the private key component $\left(U_{id},r_{U_{id}}\right)$ later. The initial value is empty, *R* = *ϕ*. For each $Att_{i}\in Z_{p}^{*}$, calculate $\frac{1}{Att_{i}}\left(\text{mod}\;p\right)$ and output

The proxy update key *UK* = (*ver*, *rk* = {*rk*_*i*_}_*i∈U*_, *L* = {*L*_*i*_}_*i∈U*_);

The proxy grant key $GK=\left(R,\frac{1}{Att_{1}},\cdots ,\frac{1}{Att_{n}},L^{*}={\left\{L_{i}^{*}\right\}}_{i\in U}\right)$.

**Do.Enc** (PP,M,T)→CT: Similar to the encryption method in [18]. It inputs the public parameter *PP*, a message *M* and an access structure T. The algorithm chooses a (*k*_*x*_−1) -degree polynomial *q*_*x*_(∙) for each node *x* in the tree T in a top-down manner. The selected polynomial *q*_*x*_(∙) must satisfy the restriction that *q*_*x*_(0) = *s* if *x* is the root node in T, otherwise *q*_*x*_(0) = *q*_*parent*(*x*)_(*indes*(*x*)), where *s* is randomly chosen from $Z_{p}^{*}$. It is worth noting that for a leaf node, because it does not have a child, so it selects constant polynomial *q*_*x*_(∙) = *q*_*x*_(0) = *q*_*parent*(*x*)_(*indes*(*x*)). Let Ψ be the set of leaf nodes in T, the ciphertext *CT* is computed as:

$$CT=\left(T,\tilde{C}=M\cdot e{\left(g,g\right)}^{\alpha s},C=g^{\beta s},\forall x\in \Psi :C_{x}=g^{q_{x}\left(0\right)T_{att\left(x\right)}},C_{x}^{*}=H{\left(att\left(x\right)\right)}^{q_{x}\left(0\right)}\right)$$

Here, the function *att*(*x*) returns the attribute associated with the leaf node *x* and *att*(*x*)∈*U*. Note that, the hash function *H*(∙) Maps an attribute to a random element of $G_{1}$, so $H\left(att\left(x\right)\right)\in G_{1}$.

**AA.KenGen** $\left(MK,U_{id},S_{U_{id}}\right)\to \left(SK_{U_{id}},A_{U_{id}},B_{U_{id}}\right)$: The key generation algorithm takes master private key *MK*, a unique user identity *U*_*id*_ and the corresponding attribute set $S_{U_{id}}$ as input. It firstly defines a user version number as the current system version number $ver_{U_{id}}=ver$. Then it chooses a random $r_{U_{id}}\in Z_{P}$, and then chooses a random *r*_*i*_∈*Z*_*P*_ for each attribute $i\in S_{U_{id}}$. It outputs the private key as:

$$SK_{U_{id}}=\left(ver_{U_{id}},D=g^{\frac{\alpha +r_{U_{id}}}{\beta }},\forall i\in S_{U_{id}}:D_{i}=g^{\frac{r_{U_{id}}}{Att_{i}}}\cdot H{\left(i\right)}^{\frac{r_{i}}{Att_{i}}},D_{i}^{*}=g^{r_{i}}\right)$$

It randomly chooses *μ* ∈ *Z*_*P*_ and set $A_{U_{id}}=\mu$ as user’s search key, and computes $B_{U_{id}}=g^{\frac{x}{A_{U_{id}}}}=g^{\frac{x}{\mu }}$ as user’s search key in CS.

**U.Dec** $\left(CT,SK_{U_{id}}\right)\to M$: Similar to the decryption method in [18]. The decryption algorithm first defines a recursive algorithm $DecNode\;\left(CT,SK_{U_{id}},x\right)$, where *x* represents a node in T. Then it is followed in a down-top manner.

- For each leaf node *x*, with *i* = *att*(*x*), if $i\in S_{U_{id}}$, it computes: $DecNode\;\left(CT,SK_{U_{id}},x\right)=\;\frac{e\left(D_{i},C_{x}\right)}{\text{e}\left(D_{i}^{*},C_{x}^{*}\right)}=e{\left(g,g\right)}^{r_{U_{id}}q_{x}\left(0\right)}$. Otherwise, it returns ⊥;
- For each interior node *x*, Lagrange interpolation is used on at least *k*_*x*_ such $e{\left(g,g\right)}^{r_{U_{id}}q_{z_{j}}\left(0\right)}$ from its children {*z*_*j*_} to calculate $\begin{array}{l} e{\left(g,g\right)}^{r_{U_{id}}q_{x}\left(0\right)} \end{array}$.

Finally, for the root node $R_{T}$ in T, let $A=e{\left(g,g\right)}^{r_{U_{id}}q_{R_{T}}\left(0\right)}=e{\left(g,g\right)}^{r_{U_{id}}s}$. The decryption can be computed by:

$$Dec\;\left(CT,SK_{U_{id}}\right)=\tilde{C}/\left(e\left(C,D\right)/A\right)=M$$

**AA.ReKenGen** (*PP*, *MK*, *γ*, Δ*L*_*γ*_, *η*, Δ*L*_*η*,_ *UK*, *GK*)→(*PP*′, *MK*′, *UK*′, *GK*′, *RK*_*γ*_): The re-encryption key generation algorithm takes public parameter *PP*, a master key *MK*, a set of attributes *γ* (the attribute in *γ* which is to be revoked for some users) and the corresponding revoked user list Δ*L*_*γ*_, a set of attribute *η* (the attribute in *η* is to be granted for some users) and the corresponding granted user list Δ*L*_*η*_, the proxy update key *UK*, and the proxy grant key *GK* as input.

If *γ* ≠ ∅, for each attribute *i* ∈ *γ*, it chooses random $Att_{i}\prime \in Z_{p}^{*}$ as the new attribute key. Then performs the following action:

- Master key update. Replaces the *Att*_*i*_ in *MK* with $Att_{i}\prime$, the rest of the parameters keeps unchanged;
- Proxy update key upodate. Replaces the *ver* in *UK* as *ver*′ = *ver* + 1, calculates $rk_{i,ver\text{'}}=\frac{Att_{i}\prime }{Att_{i}}\left(\;p\right)$ and adds to the set *rk*_*i*_ = {*rk*_*i*,0_, ⋯, *rk*_*i*,*ver*_}. For other attribute *i*∈*U* \ *γ*, adds *rk*_*i*,*ver*′_ = 1 to the set *rk*_*i*_ = {*rk*_*i*,0_, ⋯, *rk*_*i*,*ver*_} to get the updated *rk*_*i*_ = {*rk*_*i*,0_, ⋯, *rk*_*i*,*ver*_,*rk*_*i*,*ver*′_}. Then adds the identity of users whose attribute need to be revoked in Δ*L*_*γ*_ to the corresponding revocation user list *L* = {*L*_*i*_}_*i∈U*_;
- Set re-encryption key *RK*_*γ*_ = {(*i*, *rk*_*i*,*ver′*_)}_*i∈γ*_;
- Proxy grant key update. Replace the $\frac{1}{Att_{i}}$ in *GK* with $\frac{1}{Att_{i}\text{'}}\left(\text{mod }p\right)$, the rest of the parameters keeps unchanged;
- Public parameter update. Calculate $T_{i}^{\text{'}}=T_{i}{}^{rk_{i,ver^{\prime }}}=g^{Att_{i}\text{'}}$ and replace the *T*_*i*_ in *PP* with $T_{i}^{\text{'}}$, the rest of the parameters keeps unchanged.

If *η* ≠ ∅, add the identity of users in Δ*L*_*η*_ who need to be granted some attributes to the corresponding grant user list $L^{*}={\left\{L_{i}^{*}\right\}}_{i\in U}$ in proxy grant key.

**CS.ReEnc** (*γ*,*C*_*γ*_,,*RK*_*γ*_**)**→*C*_*γ*_*′*: The re-encryption algorithm takes a set of attribute *γ*, the attribute in *γ* which is to be revoked for some users, the ciphertext component *C*_*γ*_ = {*C*_*x*_}_*att(x)∈γ*_ ⊂ *CT*, the re-encryption key *RK*_*γ*_ as input.

For each attribute *i* ∈ *γ*, find the corresponding leaf node *x*, with *i* = *att*(*x*). Denote the universe of corresponding ciphertext component *C*_*x*_ set as *C*_*γ*_ = {*C*_*x*_}_*att(x)∈γ*_ ⊂ *CT*. For each attribute *C*_*x*_∈*C*_*γ*_, calculate $C_{x}\prime =C_{x}{}^{rk_{i,ver\prime }}$ and output *C*_*γ*_′ = {*C*_*x*_′}_*att(x)∈γ*_.

**PS.ReKey** $\left(U_{id},S_{U_{id}},ver_{U_{id}},D_{U_{id}},UK,\gamma \right)\to \left(S_{U_{id}}\prime ,ver_{U_{id}}\prime ,D_{U_{id}}\prime \right)$: The key regeneration algorithm takes a unique user identity *U*_*id*_ and the corresponding attribute set $S_{U_{id}}$, version number $ver_{U_{id}}$, the private key component $D_{U_{id}}={\left\{D_{i}\right\}}_{i\in \gamma }\subset SK_{U_{id}}$, proxy update key *UK*, a set of attributes *γ*, the attribute in *γ* which is to be revoked by some users as input. Then perform the following actions:

- If the user has the latest version $ver_{U_{id}}=ver$, it outputs ⊥ and exit;
- If it satisfies the condition that ∀*i* ∈ *γ* and *U*_*id*_ ∉ *L*_*i*_, denotes the attribute set $S_{U_{id}}^{\text{'}}=S_{U_{id}}$. For each $i\in S_{U_{id}}\cap \gamma$ and *U*_*id*_ ∈ *L*_*i*_, denotes the attribute set $S_{U_{id}}^{\text{'}}=S_{U_{id}}\setminus \left\{i\right\}$ and deletes the *U*_*id*_ from *L*_*i*_;
- For each $i\in S_{U_{id}}\cap \gamma$, compute $D_{i}^{*}\text{'}=D_{i}^{*}{}^{{\left(rk_{i,\left(ver_{U_{id}}+1\right)}\cdots rk_{i,ver}\right)}^{-1}}$ and replace the $D_{i}^{*}$ with $D_{i}^{*}\text{'}$. Then update the user version number as $ver_{U_{id}}\text{'}=ver$.

**PS.GrantAtt** $\left(U_{id},PP,GK,\eta \right)\to \left(\eta_{U_{id}},SK_{\eta_{U_{id}}}\right)$: The attribute grant algorithm takes a unique user identity *U*_*id*_, public parameters *PP*, proxy update key *UK*, proxy grant key *GK*, a set of attribute *η*, the attribute in *η* is to be granted for some users as input. Then perform the following actions:

- If it satisfies the condition that ∀*i* ∈ *η* and $U_{id}\notin L_{i}^{*}$, it outputs ⊥ and exit;
- Then build an attribute set $\eta_{U_{id}}$ as the grant set for user *U*_*id*_. The initial value $\eta_{U_{id}}=\emptyset$. For each *i* ∈ *η*, if $U_{id}\in L_{i}^{*}$, add attribute *i* to the attribute set $\eta_{U_{id}}$ and delete the *U*_*id*_ from $L_{i}^{*}$;
- For each $i\in \eta_{U_{id}}$, find the parameter $\left(U_{id},r_{U_{id}}\right)$ from the list *R* and randomly choose *r*_*i*_ ∈ *Z*_*P*_. Then compute $D_{i}=g^{\frac{r_{U_{id}}}{Att_{i}}}\cdot H{\left(i\right)}^{\frac{r_{i}}{Att_{i}}},D_{i}^{*}=g^{r_{i}}$, and define the grant private key component as $SK_{\eta_{U_{id}}}={\left\{D_{i},D_{i}^{*}\right\}}_{i\in \eta_{U_{id}}}$.

**DO.PreIndex** (*W*)→(*E*): The pre index generation algorithm for data owner takes the keyword set *W* = {*w*_1_,⋯, *w*_*m*_} as input.

For each keyword *w*_*i*_∈*W*, it calculates $E_{i}=H_{1}{\left(w_{i}\right)}^{l_{i}}\in G_{1}$, where $l_{i}\in Z_{p}^{*}$ is a random number.

Then, it outputs the data owner’s pre keywords index set *E* = (*E*_1_,⋯, *E*_*m*_).

**CS.Index** $\left(E,B_{U_{id}}\right)\to \left(V\right)$: The index generation algorithm by CS takes data owner’s pre keywords index set *E* = (*E*_1_,⋯, *E*_*m*_) and data owner’s search key in CS $B_{U_{id}}$ as input.

For each *E*_*i*_ ∈ *E*, it computes $V_{i}=e\left(E_{i},B_{U_{id}}\right)=e\left(H_{1}{\left(w_{i}\right)}^{l_{i}},g^{\frac{x}{A_{U_{id}}}}\right)$.

Then, it outputs CS’s index parameter set *V* = (*V*_1_,⋯, *V*_*m*_).

**DO.PostIndex** $\left(V,A_{U_{id}}\right)\to \left(I_{W}\right)$: The post index generation algorithm for data owner takes the CS’s index parameter set *V* = (*V*_1_,⋯, *V*_*m*_), his own search key $A_{U_{id}}$ and the random parameter *l*_*i*_ which he choices before as input.

- For each *V*_*i*_ ∈*V*, it computes $k_{i}=H_{2}\left(V_{i}{}^{\frac{A_{U_{id}}}{l_{i}}}\right)=H_{2}\left(e{\left(H_{1}{\left(w_{i}\right)}^{l_{i}},g^{\frac{x}{A_{U_{id}}}}\right)}^{\frac{A_{U_{id}}}{l_{i}}}\right)=\;H_{2}\left(\;e{\left(H_{1}\left(w_{i}\right),g\right)}^{x}\right)\in {\left\{0,1\right\}}^{l}$;
- set $I_{w_{i}}=\left(Q_{i},{\left[Q_{i}\right]}_{k_{i}}\right)$, where ${\left[Q_{i}\right]}_{k_{i}}$ denotes an encryption of a random number *Q*_*i*_ with the secret key *k*_*i*_ using a secure symmetric encryption algorithm, such as AES.

It builds the data owner’s post keywords index set $I_{W}=\left\{I_{w_{1}},\cdots ,I_{w_{m}}\right\}$ and outputs.

**U. PreTrap** $\left(w,A_{U_{id}}\right)\to \left(T_{w}\right)$: The pre trapdoor generation algorithm for user takes as input a keyword *w* and his own search key $A_{U_{id}}$.

It calculates the user’s pre trapdoor $T_{w}=H_{1}{\left(w\right)}^{A_{U_{id}}}$ and outputs.

**CS. PostTrap** $\left(T_{w},B_{U_{id}}\right)\to \left(k^{\prime }\right)$: The post trapdoor generation algorithm for CS input the pre trapdoor *T*_*w*_ and the user’s search key $B_{U_{id}}$ in CS.

It calculates the CS’s post trapdoor $k^{\prime }=H_{2}\left(e\left(T_{w},B_{U_{id}}\right)\right)=H_{2}\left(e\left(H_{1}{\left(w\right)}^{A_{U_{id}}},g^{\frac{x}{A_{U_{id}}}}\right)\right)=H_{2}\left(\;e{\left(H_{1}\left(w_{i}\right),g\right)}^{x}\right)$ and outputs.

**CS. Test**(*I*_*W*_, *k*′)→{1, 0}: The test algorithm by CS takes post keywords index set $I_{W}=\left\{I_{w_{1}},\cdots ,I_{w_{m}}\right\}$ and post trapdoor *k*′ = *H*_2_(*e*(*H*_1_(*w*_*i*_),*g*)^*x*^) as input. It checks the following equation holds

$$\exists I_{w_{i}}=\left(Q_{i},{\left[Q_{i}\right]}_{k_{i}}\right)\in I_{W},\text{ such that }{\left[Q_{i}\right]}_{k^{\prime }}={\left[Q_{i}\right]}_{k_{i}}$$

If the equation holds, it outputs 1. Otherwise, it outputs 0.

### B. Main Construction

#### System Setup

AA first asked about **AA.Setup** (*λ*, *U*)→(*PP*, *MK*, *UK*, *GK*) algorithm to get public parameter *PP*, master key *MK*, proxy update key *UK*, and proxy grant key *GK*. Then AA sends *PP* to CS and keeps *UK*, *GK*, *MK* secret.

#### Registration

AA to register every legal user in the system.

- Select a unique identity *U*_*id*_ and an attribute set $S_{U_{id}}$ to user;
- Call algorithm **AA.KenGen** $\left(MK,U_{id},S_{U_{id}}\right)\to \left(SK_{U_{id}},A_{U_{id}},B_{U_{id}}\right)$ to compute a private key $SK_{U_{id}}$, user’s search key $A_{U_{id}}$, and user’s search key $B_{U_{id}}$ in CS.
- Update the set $R=R\cup \left\{\left(U_{id},r_{U_{id}}\right)\right\}$ in $GK=\left(R,\frac{1}{Att_{1}},\cdots ,\frac{1}{Att_{n}},L^{*}={\left\{L_{i}^{*}\right\}}_{i\in U}\right)$;

Finally, AA transmits the tuple $\left(U_{id},A_{U_{id}},S_{U_{id}},SK_{U_{id}}\right)$ to new user, transmits *GK* to PS and transmits the tuple $\left(U_{id},B_{U_{id}},S_{U_{id}}\right)$ to CS. CS adds the new user information tuple to the users information list.

#### Establishment of Index

DO first extracts a set of keywords *W* = {*w*_1_, ⋯, *w*_*m*_} from the file to establish a keyword index.

- DO calls algorithm **DO.PreIndex**(*W*)→(*E*). It outputs DO’s pre keywords index set *E* = (*E*_1_, ⋯, *E*_*m*_). DO sends his identity *U*_*id*_ together with the pre keywords index set *E* to the CS.
- After receiving the request, CS first obtains the data owner’s corresponding $B_{U_{id}}$ according to *U*_*id*_. Then CS calls the algorithm **CS.Index** $\left(E,B_{U_{id}}\right)\to \left(V\right)$. It outputs CS’s index parameter set *V* = (*V*_1_, ⋯, *V*_*m*_). Then, CS transmits *V* to DO.
- DO inputs his own private search key $A_{U_{id}}$ and the random parameter *l*_*i*_ which he choices before, and calls algorithm **O.PostIndex** $\left(V,A_{U_{id}},l_{i}\right)\to \left(I_{W}\right)$. It outputs the DO’s post keywords index set $I_{W}=\left(I_{w_{1}},\cdots ,I_{w_{m}}\right)$.

#### File Upload

The file upload process is similar to the *D*. *Data Upload* process in literature [18]. The final document is stored in CS as Table 2.

**Table 2:** File Storage Format in CS.

*F*_*id*_	*I*_*W*_	*CT*	[*DataFile*]_*k*_

Here, *F*_*id*_ represents the file number. [*DataFile*]_*k*_ represents the encrypt file by a symmetric encryption key *k*. *I*_*W*_ represents the keywords index. *CT* represents the symmetric encryption key *k*’s ciphertext which encrypted by our proposed algorithm **Do.Enc** (PK,M=k,T)→CT. Details of file upload process can be found in *D*. *Data Upload* in literature [18].

#### Attribute Alteration

If there is no need to change any user's attributes in the system, it outputs ⊥ and exit.

If there have a set of attribute *γ* which some users be revoked, and a set of attribute *η* which some users be granted. We processes as follows.

- AA first calls the algorithm **AA.ReKenGen** (*PP*, *MK*, *γ*, Δ*L*_*γ*_, *η*, Δ*L*_*η*,_ *UK*, *GK*)→(*PP*′, *MK*′, *UK*′, *GK*′, *RK*_*γ*_) to obtain updated public parameter *PP* = *PP*′, master key *MK*, proxy update key *UK* = *MK*′, proxy grant key *GK* = *GK*′, and re-encryption key *RK*_*γ*_. Then it sends *PP*, *γ*, *RK*_*γ*_ to CS, sends *UK*, *GK* to PS and keeps *MK*, *UK*, *Gk* secret.
- On receiving *PP*, CS publishes it.
- After receive the re-encryption key *RK*_*γ*_, CS calls algorithm **CS.Enc** (*γ*, *C*_*γ*_, *RK*_*γ*_)→*C*_*γ*_′ and updates the corresponding ciphertext.

The following steps are performed when a user needs to search for a file.

#### Trapdoor Generation

First, the user set a search keyword *w*. Then he calls the algorithm **U. PreTrap** $\left(w,A_{U_{id}}\right)\to \left(T_{w}\right)$. It outputs the user’s pre trapdoor *T*_*w*_.

#### Updating Attribute and Private Key

- User *U*_*id*_ sends his parameters $\left(U_{id},S_{U_{id}},ver_{U_{id}},D_{U_{id}}\right)$ to PS.
- PS first calls algorithm **PS.ReKey** $\left(U_{id},S_{U_{id}},ver_{U_{id}},D_{U_{id}},UK\right)\to \left(S_{U_{id}}\text{'},ver_{U_{id}}\text{'},D_{U_{id}}\text{'}\right)$. It outputs updated user attribute set $S_{U_{id}}\text{'}$, version number $ver_{U_{id}}\text{'}$ and private key component $D_{U_{id}}\text{'}={\left\{D_{i}\text{'}\right\}}_{i\in \gamma }\subset SK_{U_{id}}$.
- Then PS calls the algorithm **PS.GrantAtt** $\left(U_{id},PP,GK\right)\to \left(\eta_{U_{id}},SK_{\eta_{U_{id}}}\right)$. It outputs a set of attribute $\eta_{U_{id}}$ which is to be granted to user *U*_*id*_ and the corresponding private key component $SK_{\eta_{U_{id}}}={\left\{D_{i},D_{i}^{*}\right\}}_{i\in \eta_{U_{id}}}$.
- It sets the parameters $S_{U_{id}}=S_{U_{id}}^{\text{'}}\cup \eta_{U_{id}}$.
- PS returns parameters $\left(U_{id},S_{U_{id}},ver_{U_{id}}\text{'},D_{U_{id}},SK_{\eta_{U_{id}}}\right)$ to user and send $\left(U_{id},S_{U_{id}}\right)$ to CS.
- User updates his own parameters $S_{U_{id}}$ and $SK_{U_{id}}$. CS updates tuple $\left(U_{id},B_{U_{id}},S_{U_{id}}\right)$ for user *U*_*id*_’s attribute in the users information list.

#### Search the File by CS

*U*_*id*_ sends his trapdoor $T_{w}=H_{1}{\left(w\right)}^{A_{U_{id}}}$ and his unique identity *U*_*id*_ to CS. CS performs the following action.

- according to user's identity *U*_*id*_ CS finds the corresponding user attribute set $S_{U_{id}}$ and user’s search key $B_{U_{id}}$ in CS from user information list tuple $\left(U_{id},B_{U_{id}},S_{U_{id}}\right)$.
- For the trapdoor $T_{w}=H_{1}{\left(w\right)}^{A_{U_{id}}}$ and user’s search key $B_{U_{id}}$, CS calls algorithm **CS. PostTrap** $\left(T_{w},B_{U_{id}}\right)\to \left(k^{\prime }\right)$. It outputs CS’s post trapdoor *k*′.
- According to attributes set $S_{U_{id}}$, CS has to search documents by performing the *Step3*: *search the data by the cloud server* process in literature [18].
- For all documents in the files collection that the user can decrypt, matches the keyword trapdoor with the keywords index, to find the user's interested files in the document.
- According to the search parameter *k*′, it runs algorithm **CS. Test**(*I*_*W*_, *k*′)→{1, 0}. If the output is 1, it returns the corresponding *CT* and {*DataFile*}_*k*_ to the user.

#### File Decryption

User to decrypt ciphertext by calling decryption algorithm **U.Dec** $\left(CT,SK_{U_{id}}\right)\to M=k$. User to further decrypt the symmetric ciphertext {*DataFile*}_*k*_ to get the document {*DataFile*}.

Similar to literature [18], we can also perform most of the calculation process by PS.

#### User Revocation

Our scheme by removing user’s search key $B_{U_{id}}$ in CS to achieve user identity revocation. Because if CS to remove user's $B_{U_{id}}$, the user will not be able to successfully search files.

### C. Flowchart of Our Proposed Scheme

**Fig 2:**
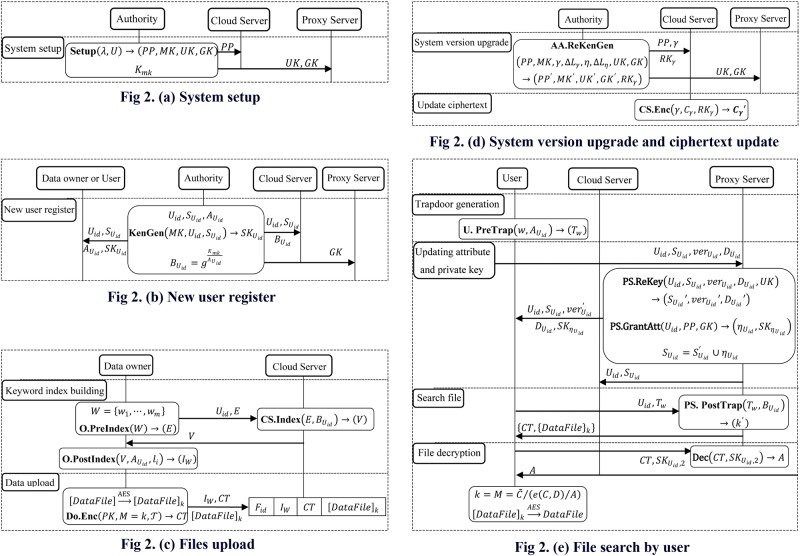
Flowchart of Our Proposed Scheme.

We set a legitimate user *U*_*id*_ first as a data owner to upload their own data, and then as a user access to the content of the interest files. The flowchart of our Fig 2(a), 2(b), 2(c), 2(d) and 2(e) respectively gives the process of system setup, new user registration, file upload, system version upgrade and ciphertext update, file search by user of our scheme.

## V. Security

### A. Security Analysis

First of all, we analyze our scheme. There are six entities in the project: AA, PS, CS, DO, U.

AA is responsible for establishing the program, and we set it to be fully trusted.

Our PS is subordinate to authority. In order to reduce the computing load of AA and ensure the efficiency of program, we grant a lot of functions to PS. One of the most important functions is to grant user's attribute and the corresponding private key, which makes our PS must be trusted. If we think about the problem of malicious PS, we have to leave granted rights to AA. Only AA can execute the private key grant rights, which can improve the security of scheme to a certain extent, but it will reduce efficiency of scheme. It introduces a new model. We aim to study the integrity of our proposed scheme, which has not made a number of analysis to this new model.

DO has no difference from other users in set the private key in addition to having the data files to be uploaded. It is to say that keyword search process of DO is equivalent to a general user. Due to the users access permissions are different according to their own attribute set. Some users may want to access more data files beyond their access permissions. So one of the attack models we consider is derived from a malicious user. He may also be a legitimate user. We will show that our scheme is secure against this attack model.

CS is an outsourced server. As in most articles, we assume CS is “curious-but-honest” [13]. It is to say that CS is curious about the encrypted data contents or the received messages, but will execute correctly the proposed tasks. It might be interested in the content of user search, so another attack models we consider is derived from a malicious CS.

### B. Attack Model 1 (IND-sCP-CPA Security Model)

The adversary A is assumed to be an outsider attacker including the users in the system.

Through the establishment of a security game model, we reduce the security of our scheme to Bethencourt’s scheme [3]. According to the proof of reference [3] in its appendix A (Bethencourt’s scheme is IND-sCP-CPA secure), our scheme is also IND-sCP-CPA secure in the attack model 1. The proof procedure is as follows.

**Theorem 5** Suppose that the Bethencourt’s scheme is IND-sCP-CPA secure, then our scheme is also IND-sCP-CPA secure in the attack model 1.

**Proof.** We consider a simulator S' of Bethencourt’s scheme, a simulator S of our scheme and a polynomial-time adversary A of our scheme. It is noteworthy that the simulator S of our scheme has another identity who is also an adversary A' of Bethencourt’s scheme. Suppose that A of our scheme is able to distinguish a valid ciphertext from a random element with advantage *ε*. We build a simulator S (namely A' of Bethencourt’s scheme) that can attack Bethencourt’s scheme with the same advantage. The simulation proceeds as follows.

**Int.** A declares an access structure $T^{*}$, which he wishes to be challenged upon. The simulator S declares the same access structure.

**Setup.** The simulator S' takes a security parameter *λ* and runs the **Setup** algorithm of Bethencourt’s scheme. It gives the public parameter $PP\prime =\left(G_{1},g,g^{\beta },e{\left(g,g\right)}^{\alpha }\right)$ to the simulator S. After receive the public parameter *PP*′, the simulator S randomly chooses $Att_{i}\in Z_{p}^{*}$ for ∀*i* ∈*U* as the attribute parameter. Then for all *i* ∈ *U*, *j* = 1, 2, ⋯, *ver*, it randomly chooses $rk_{i,j}\in Z_{p}^{*}$ and the public parameter generated as follows.

$$PP=\left(G_{1},g,g^{\beta },e{\left(g,g\right)}^{\alpha },T_{1}=g^{Att_{1}},\cdots ,T_{n}=g^{Att_{n}}\right)$$

$$MK\prime =\left(ver,Att_{1},\cdots ,Att_{n},rk={\left\{rk_{i}\right\}}_{i\in U}\right)$$

Then, it send the public parameter *PP* to A.

**Phase 1.** A adaptively issues polynomial following queries.

- ***private key query***. A submits a set of attributes *S* where *S* does not satisfy the access structure $T^{*}$ to the simulator S. The simulator S submits the same attributes to the simulator S'. Then the simulator S can get the $SK\prime =\left(D=g^{\alpha +r/\beta },\forall i\in S:D_{i}\prime =g^{r}H{\left(i\right)}^{r_{i}},D_{i}^{*\prime }=g^{r_{i}}\right)$ from the simulator S'. The simulator S calculates as follows. For all *i* ∈ *S*, $D_{i}={\left(D_{i}\prime \right)}^{1/Att_{i}}$. The A is given private key $SK=\left(D=g^{\alpha +r/\beta },\forall i\in S:D_{i}={\left(D_{i}\prime \right)}^{1/Att_{i}},D_{i}^{*}=D_{i}^{*\prime }\right)$.
- ***update private key query***. A is allowed to issue queries for update private key *SK* for the attribute in *γ* which is to be revoked for some users. A submits a part of the private key $\hat{SK}=\left(\forall i\in S:D_{i},D_{i}^{*}\right)$ he asked before where *S* ∩ *γ* ≠*ϕ*.
  1. For all attribute *i* ∈ *γ*, the simulator S randomly chooses $rk_{i}\in Z_{p}^{*}$ and maintains the update key {*rk*_*i*_}_*i∈γ*_.
  2. For all attribute *i*∈*S*∩*γ* in $\hat{SK}$, the simulator S calculates $D_{i}\prime =D_{i}^{\frac{1}{rk_{i}}}$ and keeps other parameters unchanged. Then returns the update private key $\hat{SK\prime }$ to A.

**Challenge.** A submits two messages *M*_0_ and *M*_1_ on which he wishes to be challenged upon. S outputs the same messages to S'. Then S' flips a random coin *b* ∈ {0, 1}, and encrypts *M*_*b*_ with access structure $T^{*}$. S' sends the ciphertext $CT\prime =\left(T^{*},\tilde{C}=M_{b}\cdot e{\left(g,g\right)}^{\alpha s},C=g^{\beta s},\forall x\in \Psi :C_{x}\prime =g^{q_{x}\left(0\right)},C_{x}^{*\prime }=H{\left(att\left(x\right)\right)}^{q_{x}\left(0\right)}\right)$ to S. The simulator S calculates CT* as follows. For all *x* ∈ Ψ, $C_{i}={\left(C_{x}\prime \right)}^{T_{att\left(x\right)}}$.A is given attribute key $CT^{*}=\left(T^{*},\tilde{C}=M\cdot e{\left(g,g\right)}^{\alpha s},C=g^{\beta s},\forall x\in \Psi :C_{i}={\left(C_{x}\prime \right)}^{T_{att\left(x\right)}},C_{x}^{*}=C_{x}^{*\prime }\right)$.

**Phase 2.** Repeated phase 1 adaptively.

**Guess.** A submits a guess *b*′ of *b*. S outputs the guess *b*′ to indicate that it was given the *CT*′. If A is able to distinguish the valid ciphertext with advantage $\left|\text{Pr}\left[b^{\prime }=b\right]-\frac{1}{2}\right|=\varepsilon$. We build the simulator S that can distinguish the valid ciphertext in Bethencourt’s scheme with the same advantage.

### C. Attack Model 2 (IND-CKA Security Model)

The adversary A is assumed to be CS.

We will prove that our scheme of semantic security for keywords trapdoor. Notice that in the search process of our scheme the public parameter is *g*, the private key of the user is $A_{U_{id}}=\mu$, the private key of CS is $B_{U_{id}}=g^{x/\mu }$, the master private key of the attribute authority is *K*_*mk*_ = *x*. We assume that A is a malicious CS, then the public parameters related to the search process that it can get are $\left(g,B_{U_{id}}=g^{x/\mu }\right)$.

**Theorem 6.** Assuming the BDH (Bilinear Diffie-Hellman) assumption was founded. Then our scheme has the IND-CKA security in the random oracle model.

**Proof.** We consider a chosen-keywords-attack polynomial-time adversary A and a simulator S.

Suppose that A is able to correctly distinguish keywords with advantage *ε*. We build a simulator S that can solve the BDH problem with at least $\varepsilon \prime =2\varepsilon /\hat{e}q_{T}q_{H_{2}}$, Where *ê* is the base of the natural logarithm, *q*_*T*_ > 0 is the number of pre trapdoor queries, $q_{H_{2}}>0$ is the number of hash queries.

**Int.** The simulator S runs A and receives a BDH challenge. It first chooses two multiplicative cyclic groups $G_{1},G_{2}$ of prime order *p* and a bilinear map $e:G_{1}\times G_{1}\to G_{2}$. S is given $g\in G_{1}$, as well as *u*_1_ = *g*^*α*^, *u*_2_ = *g*^*β*^, *u*_3_ = *g*^*γ*^ for some random $\alpha ,\;\beta ,\;\gamma \in {\mathbb{Z}}_{p}$. S’s goal is to get $\text{e}{\left(g,g\right)}^{\alpha \beta \gamma }\in G_{2}$.

**Setup.** The simulator S announces the public parameter $\left(g,B_{U_{id}}=u_{3}\right)$ with the implicit assumption that $K_{mk}=x=\beta \cdot \;\gamma ,\;\;A_{U_{id}}=\mu =\beta$. According to the above settings, we can calculate that $B_{U_{id}}=g^{x/\mu }=g^{\gamma }=u_{3}$.

**Phase 1.** A adaptively issues polynomial following queries.

- ***H***_**1**_**-Query:** A can always ask the random oracle *H*_1_ of any keyword *w*_*i*_ ∈ {0, 1}*. S answers the questions of A and records the results of each answer.
  If A submits a keyword *w*_*i*_ ∈ {0, 1}* that has not been asked, S does the following.
  1. S generates a random coin *c*_*i*_ ∈ {0, 1}, so that Pr[*c*_*i*_ = 0] = 1/(*q*_*T*_ + 1).
  2. S picks a random element $a_{i}\in Z_{p}^{*}$. If the coin *c*_*i*_ = 0, S computes $h_{i}=u_{1}\cdot g^{a_{i}}\in G_{1}$. If *c*_*i*_ = 1, S computes $h_{i}=g^{a_{i}}\in G_{1}$.
  3. S adds the tuple (*w*_*i*_, *h*_*i*_, *a*_*i*_, *c*_*i*_) to the list *H*_1_-list, and returns *H*_1_(*w*_*i*_) = *h*_*i*_ to A.
    If A submits a query *w*_*i*_ that has been asked, then S finds the tuple (*w*_*i*_, *h*_*i*_, *a*_*i*_, *c*_*i*_) in the *H*_1_-list and responds $H_{1}\left(w_{i}\right)=h_{i}\in G_{1}$ to A.
- ***H***_2_-**Query:** A can always ask the random oracle *H*_2_ of any $t_{i}\in G_{2}$. S answers the questions of A and records the results of each answer.
  If A submits a $t_{i}\in G_{2}$ that has not been asked. S chooses a random number *H*_2_(*t*_*i*_) = *V*_*i*_ ∈ {0, 1}^log *p*^ and adds the tuple (*t*_*i*_, *V*_*i*_) to the list *H*_2_-list. Then it returns *H*_2_(*t*_*i*_) = *V*_*i*_ to A.
  If A submits a query *t*_*i*_ that has been asked, then S finds the tuple (*t*_*i*_, *V*_*i*_) in the *H*_2_-list and responds *H*_2_(*t*_*i*_) = *V*_*i*_ to A.
- **Pre-trapdoor queries:** A can also ask the pre-trapdoor of any keyword *w*_*i*_ ∈ {0, 1}*. S answers the questions of A as folloes.
  1. For a keyword *w*_*i*_ ∈ {0, 1}*, S executes *H*_1_-Query to get a tuple (*w*_*i*_, *h*_*i*_, *a*_*i*_, *c*_*i*_).
  2. If *c*_*i*_ = 0, S declares a failure and ends the game.
  3. If *c*_*i*_ = 1, $\;H_{1}\left(w_{i}\right)=h_{i}=g^{a_{i}}\in G_{1}$. S generates $T_{w_{i}}=u_{2}{}^{a_{i}}={\left(g^{a_{i}}\right)}^{\beta }$ and returns $T_{w_{i}}$ to A as response for the query.

**Challenge.** A submits two keywords *w*_0_ and *w*_1_ where the keywords *w*_0_ and *w*_1_’s trapdoor had not asked by A.

- S initiates *H*_1_-Query twice to obtain $h_{0},h_{1}\in G_{1}$, where *H*_1_(*w*_0_) = *h*_0_, *H*_1_(*w*_1_) = *h*_1_. If *c*_0_ = 1 and *c*_1_ = 1, then S reports a failure and terminates.
- Otherwise, we know at least one of *c*_0_ and *c*_1_ is equal to 0. If *c*_0_ = 0 and *c*_1_ = 0, S picks randomly a bit *b* ∈ {0, 1}.
- S picks a random element *k* ∈ {0, 1}^*log p*^, and return {*u*_3_, *k*} to A as a response, where *k* imitates the post trapdoor in our proposed scheme. Note that, if A has an advantage in answer the above question. We have the implied settings:

$$\begin{array}{ll} k & =H_{2}\left(e\left(T_{w},\;B_{U_{id}}\right)\right)\\ & =\;H_{2}\left(e\left(\;H_{1}{\left(w_{i}\right)}^{\beta },\;u_{3}\right)\right)\\ & =\;H_{2}\left(e\left(\;H_{1}{\left(w_{i}\right)}^{\beta },\;u_{3}\right)\right)\\ & =\;H_{2}\left(e\left(g^{\left(\alpha +a_{b}\right)\;\beta },\;g^{\gamma }\right)\right)\\ & =\;H_{2}\left(e{\left(g,\;g\right)}^{\;\beta \gamma \left(\alpha +a_{b}\right)}\right) \end{array}$$

**Phase 2.** Repeated phase 1 adaptively.

**Guess.** A submits a guess *b*′ of *b*. If *b*′ = *b*, A wins the game and break our scheme.

#### Correctness Analyses

In the above simulation scheme, if the adversary can break the game and distinguish the keyword with a non negligible probability that means that the random element *k* it chooses is $H_{2}\left(e{\left(g,\;g\right)}^{\;\beta \gamma \left(\alpha +a_{b}\right)}\right)$. Then S can compute that $\frac{k}{u_{2}u_{3}g^{a_{b}}}=\text{e}{\left(g,g\right)}^{\alpha \beta \gamma }$ which means it solves the DDH problem.

#### Probability Analyses

We can prove that if A can win the game with a non negligible probability *ε*, then S can solve the BDH problem with the probability at least $2\varepsilon /eq_{T}q_{H_{2}}$. That process in detail in [36].

Because of the BDH assumption that the BDH problem is difficult, so the probability $2\varepsilon /eq_{T}q_{H_{2}}$ is negligible. That is, our scheme is safe.

**Fig 3:**
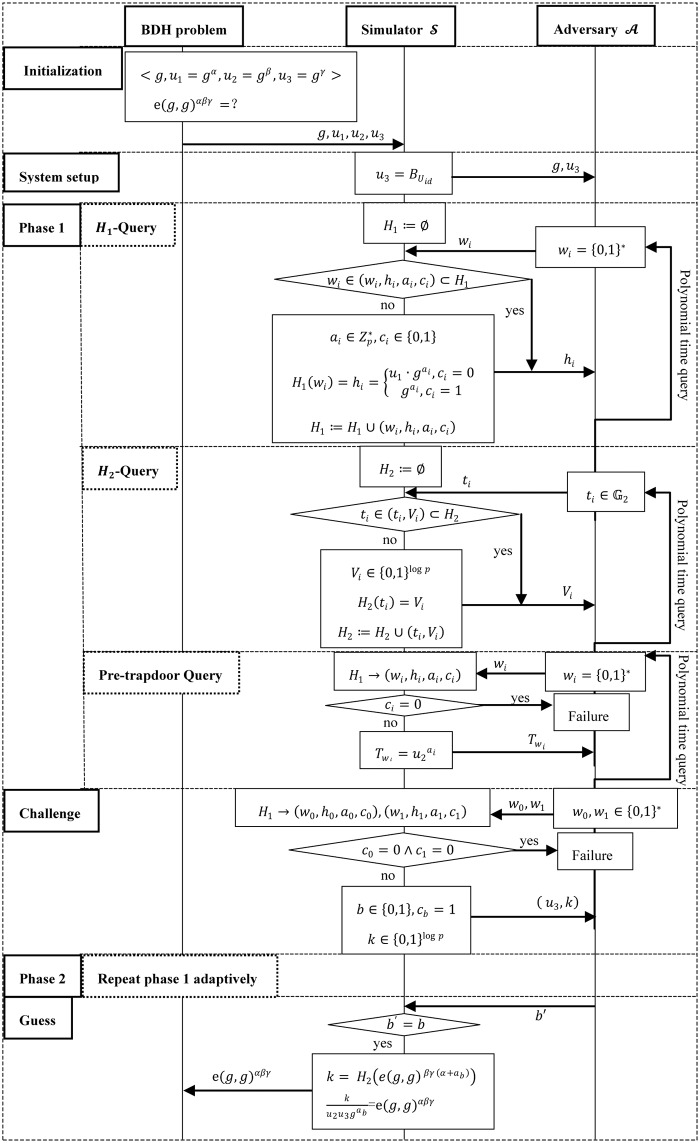
Flowchart of attack model 2.

Taking attack model 2 (a selected keyword attack model from the cloud server) as an example, we give the specific flow chart of the game process in Fig 3.

## VI. Performance Analysis and Comparison

### A. Performance Analysis

**The time complexity of our scheme.** In the **Setup** phase, a public parameter and master key are generated. At this stage, the total number of attributes is defined as n. An exponentiation operation in $G_{1}$ or $G_{2}$ is defined as *e*. A pairing operation is defined as *p*. The time complexity of generating *PP*, *MK*, *UK*, *GK* is (2 + 2*n*)e + *p*, 0, 0, *ne* respectively. We calculate the total time complexity of **Setup** is (2 + 3*n*)*e* + *p*.

In algorithm **Encrypt** for *d*_2_ number of attributes that associated with access structure. In order to compute *CT*, the user needs to run (2+2*d*_2_)*e* + *p* operations. So the time complexity of Encrypt is (2 + 2*d*_2_)*e* + *p*.

When generating the private key for a user with number of attributes *d*_1_, AA needs to run (2 + 3*d*_1_) *e* addition operations in order to compute *SK*. So the time complexity of **KeyGeneration** algorithm is (2 + 3*d*_1_) *e*.

In algorithm **re encryption** for *d*_3_ number of attributes that ciphertext needs to update, CS needs to run *d*_3_e addition operations in order to update ciphertext component. So the time complexity of **re encryption** algorithm is *d*_3_e.

In algorithm **private key re generation** for *d*_4_ number of attributes that a user needs to update, the PS needs to run *d*_4_e addition operations in order to update ciphertext component. So the time complexity of **private key re generation** algorithm is *d*_4_e.

In algorithm **attribute grant** for *d*_3_ number of attributes that a user needs to granted, PS needs to run *d*_3_e addition operations in order to compute the corresponding *SK*. So the time complexity of **attribute grant** algorithm is *d*_3_e.

In algorithm **pre trapdoor** generation for a keyword *w*, the user needs to run an exponentiation operation in order to hide the keyword. So the time complexity of **pre trapdoor** algorithm is *e*.

In algorithm **post trapdoor** generation for CS, the user needs to run a pairing operation. So the time complexity of **post trapdoor** algorithm is *p*.

In algorithm **decryption** for *d*_6_ number of user’s attributes satisfying an access structure, the data owner needs to run 2*e* + (1 + *d*_6_)*p* addition operations in order to compute the message *M*. So the time complexity of **decryption** algorithm is 2*e* + (1 + *d*_6_)*p*.

### B. Comparison

We compare the computational complexity of our scheme with the existing schemes presented in [3, 13, 18] for the specific process in Table 3.

**Table 3:** Comparisons of Computational Complexity.

Schemes	[3]	[18]	[13]	Ours
*PP*	2e+p	2e+p	(2 + 2*n*)e+p	(2 + 2*n*)e+p
*SK*	(2 + 3*d*_1_)e	(2 + 3*d*_1_)e	(2 + *d*_1_)e	(2 + 3*d*_1_)e
*CT*	(2 + 2*d*_2_)e+p	(2 + 2*d*_2_)e+p	(1 + 2*d*_2_)e+p	(2 + 2*d*_2_)e+p
*CT* Update	✘	✘	*d*_3_e	*d*_3_e
*SK* Update	✘	✘	*d*_4_e	*d*_4_e
*SK* Grant	✘	✘	*d*_3_e	*d*_3_e
*T*_*r*_ by user	✘	e	✘	e
*T*_*r*_ by CS	✘	p	✘	p
*DK*	2e+(1 + *d*_6_)p	2e+(1 + *d*_6_)p	(1 + 2*d*_6_)e+(2 + 2*d*_6_)p	2e+(1 + *d*_6_)p

e: an exponentiation operation in $G_{1}$ or $G_{2}$;

p: a pairing operation;

*d*_1_: number of attributes that a user possess;

*d*_2_: number of attributes that associated with access structure;

*d*_3_: the number of attributes that ciphertext need to update;

*d*_4_: number of attributes that a user needs to update;

*d*_5_: number of attributes that a user needs to grant;

*d*_6_: the number of user’s attributes satisfying an access structure;

✘: there is no corresponding function or process in the literature.

### C. Simulation and Evaluation

**Fig 4:**
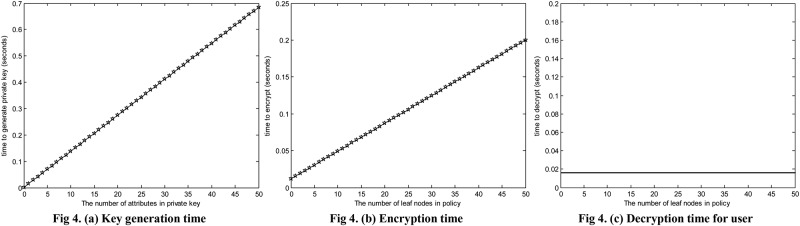
The performance of CP-ABE.

In order to evaluate the performance of our CP-ABE construction, we test the runtime of the core algorithms Key Generation, Encryption and Decryption by user with different number of attributes. Fig 4 shows the test result. The implementation uses the Pairing Based Cryptography (PBC) library [37]. We can clearly see from Fig 4 that the key generation time and the encryption time increase with the number of attributes linearly, and the decryption time keeps constant. This result is in agreement with our time complexity analysis in section Security and Performance analysis.

## VI. Application

Our scheme is well suited for applications in cloud computing environments. Take search engine file management system for example. Firstly, users can become legitimate users by registered members. After the successful landing of legitimate users, users can not only search for documents of interest, but also upload local files to server.

On the one hand because of the excessive number of users and documents, the system through the outsourcing of data files to a CS.

On the other hand because the grade of membership system of the operating construction, making part of the document can only download by some VIP members. In order to facilitate the management of the system, the system can set up an interior PS to help manage user membership grade and duration.

Ordinary users can become VIP users by way of payment. The process of granting the VIP attribute does not require the system upgrade and the update of the ciphertext. AA only to issue a grand command to the PS. When a user access, PS verify that the user is required to grant the attribute according to the identity. To user who needs to be granted attributes, PS will grant the attribute and private key to the corresponding user in time.

Once the VIP attribute is invalid or expires, AA will update the system in a timely manner and send update command to PS.

## VII. Conclusion

In this paper, we propose an efficiently multi-user searchable encryption with attribute revocation and grant function for cloud storage.

- In the first scenario, we propose a CP-ABE scheme with attribute revocation and grant. Our scheme can not only support a single user attribute revocation or grant, but also to some users to grant or revoke a set of attributes.
- In the second scenario, we propose a multi user search scheme based on a single keyword. As we focus on the user attribute update instead of the keyword search in this paper. Aiming at the problem of conjunctive keyword search is a direction that we continue to research.
- In addition, the lazy update of the user's attribute and the private key increases the efficiency of the scheme.
- Since PS in our scheme has the permissions granted attributes, in order to prevent a malicious PS to the user to grant a new attribute, we ask our PS must be honest and strict implementation of the tasks assigned by attribute authority. In other words, PS in strict accordance with the grant list to verify whether the user needs to grant attributes. Aiming at the problem of PS malicious attacks is another direction that we continue to research.

## Supporting Information

S1 Appendix (DOCX)
