# Supplementary material for: Efficiently Multi-User Searchable Encryption Scheme with Attribute Revocation and Grant for Cloud Storage
Source: PLoS One. 2016 Nov 29;11(11):e0167157. doi: 10.1371/journal.pone.0167157 (PMC5127603; doi:10.1371/journal.pone.0167157)
Supplement: S1 Appendix — (DOCX) [file pone.0167157.s001.docx]

**S1 Appendix**

The experimental part of this paper refers to the results in the Pairing Based Cryptography (PBC) library.

**The runtime of cryptographic operations**

| Operating | Ad | Ne | Mu | In | Ex | Add | Neg | PM | Mul | Inv | Exp | P |
| --- | --- | --- | --- | --- | --- | --- | --- | --- | --- | --- | --- | --- |
| Times/ms | 0.001 | 0.000 | 0.001 | 0.004 | 0.067 | 0.038 | 0.001 | 8.006 | 0.013 | 0.041 | 1.882 | 16.064 |

^1^Ad: an addition operation in $Z_{P}$;

^2^Ne: the inverse in addition operation in $Z_{P}$;

^3^Mu: a multiplication operation in $Z_{P}$;

^4^In: the inverse in multiplication operation in $Z_{P}$;

^5^Ex: an exponentiation operation in $Z_{P}$;

^6^Add: an addition operation in $\mathbb{G}_{1}$;

^7^Neg: the inverse in addition operation in $\mathbb{G}_{1}$;

^8^PM: a point multiplication operation in $\mathbb{G}_{1}$;

^9^Mul: a multiplication operation in $\mathbb{G}_{T}$.

^10^Inv: the inverse in multiplication operation in $\mathbb{G}_{T}$.

^11^Exp: an exponentiation operation in $\mathbb{G}_{T}$.

^12^P: an bilinear pairings operation in $\mathbb{G}_{T}$.
